# Extension of French vaccination mandates: from the recommendation of the Steering Committee of the Citizen Consultation on Vaccination to the law

**DOI:** 10.2807/1560-7917.ES.2018.23.17.18-00048

**Published:** 2018-04-26

**Authors:** Daniel Lévy-Bruhl, Jean-Claude Desenclos, Sylvie Quelet, François Bourdillon

**Affiliations:** 1 Santé Publique France, Saint-Maurice, France

**Keywords:** France, vaccines and immunisation, mandatory vaccination

## Abstract

On 4 December 2017, French parliamentarians passed a law extending the vaccination mandates for children up to 2 years of age from three vaccinations (against diphtheria, tetanus and poliomyelitis) to 11 by adding vaccinations against pertussis, *Haemophilus influenza b* (Hib), hepatitis B, pneumococcal diseases, meningococcal C diseases, measles, mumps and rubella. This vote follows a recommendation made by the Steering Committee of the Citizen Consultation on Vaccination that took place in 2016. The law applies to all children born after 1 January 2018. Parents who do not fulfil the mandate will not be fined but non-vaccinated children will not be admitted to any collective child services such as nurseries or schools. No exemption other than for medical reasons will be considered. Here we describe the historical background of this evolution and its main epidemiological, sociological and policy drivers. They mainly refer to insufficient vaccine coverage, persistence of a preventable burden for some diseases and growing vaccine hesitancy in the French population. We also discuss some of the challenges and conditions of success.

## Background

Up to 2018, in France, three vaccinations, namely those against diphtheria, tetanus and poliomyelitis (DTP) were mandatory for children up to 18 months of age. This was a legacy from the past as those mandates have been introduced between 1938 (diphtheria) and 1964 (poliomyelitis) with the intention to ensure a full and free access to those vaccinations for all infants, irrespective of their socio-cultural or socioeconomic background rather than as a coercive measure [1]. Vaccines introduced later in the routine vaccination programme for children were only recommended, as it was considered that there were no geographical, financial or sociological restrictions on access to those vaccinations, and consequently respective mandates were not required.

The coexistence of these different legal statuses for infant vaccinations in the routine schedule was, however, identified in several recent official reports as a weakness of the vaccination programme and the reports consistently advocated the harmonisation of the legal statuses under the National Immunisation Programme [1,2].

The most recent report, produced by a member of the French Parliament in 2015, upon the request of the Prime Minister, concluded that ‘the *status quo* is no longer possible’, and recommended a public debate on that issue [3]. This recommendation was an important driver of the decision in January 2016, of the Minister of Health in charge at the time, to organise a Citizen Consultation on vaccination. An 18-member steering committee was set up in March 2016 with the mission to report to the Minister before the end of the year. It was composed of equal numbers of civil society representatives, social scientists, and immunisation experts. They based their conclusions on 44 hearings, an Internet platform that collected more than 10,000 contributions of the public and the reports of two juries, one made up of health professionals, and another one consisting of members of the general population (http://concertation-vaccination.fr). The French National Public Health Agency (Santé publique France, St Maurice) brought scientific, logistical and administrative support to the project.

## The recommendation of the Steering Committee of the Citizen Consultation

The Committee made a wide range of recommendations to restore the population’s confidence in vaccination, to facilitate access to vaccines and vaccination services and to increase vaccine coverage [4]. Concerning the legal status of vaccinations, the Committee recommended a temporary extension of the mandates to all vaccines to be administered to all children during their first 2 years of life. Why did it chose this option rather than lifting the DTP vaccination mandates which was the alternative option to harmonise the status of childhood vaccinations? This choice may seem in contradiction with a French Law from 2002 that recognises the principle of a ‘free and informed consent to any medical act and treatment’ for everyone and with the growing societal demand for more empowerment and freedom of choice regarding healthcare [5]. As documented below, the decision was mainly based on epidemiological and sociological data and evidence provided by Santé publique France to the Citizen Consultation.

## Epidemiological drivers of the recommendation in favour of extending the vaccination mandates

In France, vaccine coverage data indicate an almost complete coverage of infants with vaccines already mandatory (DTP) and for vaccines combined with them in a single multivalent product (pertussis, *Haemophilus influenza* b (Hib)). The latest figures for children aged 2 years in 2015, indicate a coverage of 99% for DTP and pertussis and 98% for Hib. However, coverage for hepatitis B at 2 years of age was 88%. Several surveys suggested that this 10% lower coverage is likely to reflect a reluctance of ca 10% of parents to immunise their child against hepatitis B as an infant [6]. Indeed not immunising an infant against hepatitis B requests an active choice in favour of a pentavalent vaccine without hepatitis B, instead of the hexavalent vaccine recommended in the French immunisation schedule. Coverages for recommended vaccines in the second year of life (against measles-mumps-rubella and meningococcal C disease (menC) vaccines) remain suboptimal [7].

## Measles vaccine coverage

**Figure 1:**
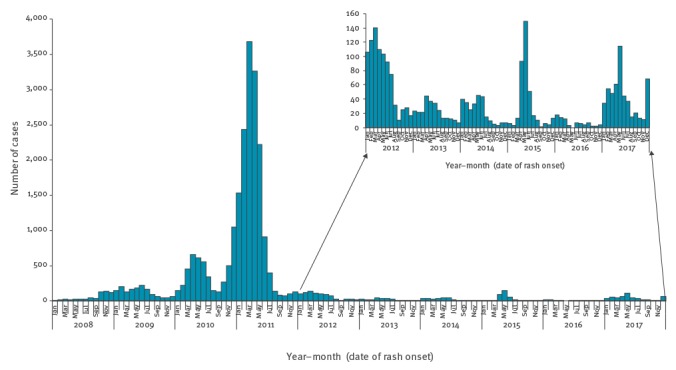
Measles cases per month, mandatory reporting, France, 2008–2017 (n = 24,559)

For the first dose of measles vaccine, coverage has levelled off at around 90% and for the second dose it has been below 80%, with an insufficient catch-up in older children [7,8]. The resulting levels of immunity in children and young adults are below the measles herd immunity threshold of 95%. A sero-epidemiological study conducted in 2009–10 showed that 8% of subjects aged 6 to 29 years were susceptible to measles [9]. After a long period of very low circulation of the virus, a large outbreak occurred in 2008–12, as anticipated by modelling [10]. Taking into account an estimated under-notification rate of ca 50%, more than 40,000 cases of measles occurred during this large outbreak (Figure 1). Thirty-one encephalitis cases, and 10 deaths were reported. Of the latter, seven occurred in individuals with contraindication to vaccination due to either a disease or treatment-related immunosuppression. Since 2017, a new resurgence of measles has been observed in France with more than 1,800 cases notified between January 2017 and March 2018, including four encephalitis cases and two deaths, one in a non-vaccinated adolescent and one in a non-vaccinated young adult. A recent analysis of the French death certificates database showed that since 2017, measles had actually caused 21 deaths (data not shown). Most of the measles-related deaths would have been avoided if the herd immunity threshold had been reached.

## Coverage of vaccination against MenC

**Figure 2:**
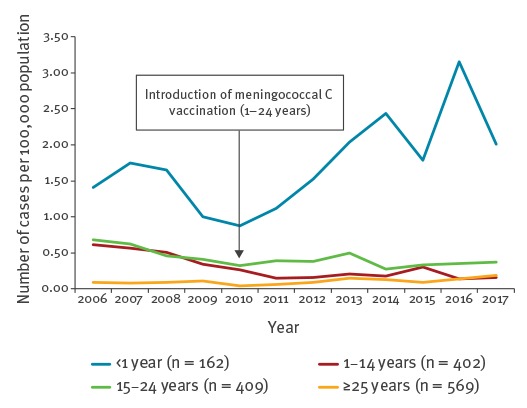
Invasive meningococcal C disease cases per year, mandatory reporting, France, 2006–2017 (n = 1,542)

Vaccination against MenC was introduced in the immunisation schedule in 2010 as a single dose at 12 months of age with a recommendation of a catch-up by 24 years of age. The strategy assumed that a high coverage in this age group would induce a herd immunity that would protect children under one year, without having to add three extra doses to the infant immunisation schedule. This strategy failed and the incidence of invasive meningococcal disease (IMD) due to MenC in children under one year increased between 2010 and 2016 in the context of a new epidemic cycle, despite the vaccination recommendation (Figure 2). The 2016 vaccination coverage for MenC was only 78% at 2 years of age with a rapid decrease as age increased (36% in pre-adolescents and 26% in adolescents) [7]. Between 2011 and 2017, 339 cases and 31 deaths were reported in unvaccinated 1–24 year-olds; an additional 493 cases and 74 deaths occurred in those under 1 or above 25 years of age. Experience in the Netherlands demonstrated that with a coverage of 94% in 1 to18 year-olds, achieved through a massive catch-up campaign in 2002, MenC was virtually eliminated as of 2004 [11]. We thus concluded that a large proportion of the more than 800 cases and 100 deaths seen in France since the introduction of MenC immunisation could have been avoided through either direct protection or herd immunity if the coverage had reached similar levels as in the Netherlands.

## Sociological drivers of the recommendation in favour of extending the vaccination mandates

Repeated quantitative and qualitative social studies using the same methodology over time (Health barometers) brought additional arguments, that lifting current vaccination mandates may not be appropriate in the French context. In 2010, the proportion of the population in disfavour (‘very unfavourable’ and ‘somewhat unfavourable’) of vaccination increased from less than 10% to almost 40%. This increase has been shown to mainly result from the controversy about the influenza A(H1N1) pandemic vaccination campaign of 2009 [12]. Although the situation has improved since, in the 2016 Health Barometer survey, almost 25% of respondents were still not or little favourable to vaccination in general [13]. In addition, 13% answered that, if DTP vaccination became only recommended, they would either not (4%) or probably not (9%) vaccinate their child. Although these answers only reflected intentions in a hypothetical scenario, the Steering Committee considered them as an alert pledging in disfavour of the lifting of the mandates for DTP.

The Committee was, however, sensitive to the risk that a mandatory measure for all infant vaccines could fuel vaccine hesitancy. Another qualitative survey commissioned by Santé publique France in spring 2016, explored the perception of mandatory vaccination in the French population. It showed that non-mandatory vaccinations were perceived as optional and that their usefulness, effectiveness and safety were often questioned. In contrast, the mandatory status had a positive effect on participants’ perception of the importance of vaccination [14].

## From the recommendation to the new policy

Based on the report of the Committee, the newly appointed Minister of Health announced in July 2017 her intention to draft a law extending the mandates for all routine vaccinations for children up to 2 years of age. The law, which applies to all children born after 1 January 2018, was passed in the French parliament in early December 2017 and the application decree was published on 25 January 2018 [15]. The mandates extend the already existing impossibility for parents to have their unvaccinated child attending daycare centers or schools from three (DTP) to 11 vaccinations by adding vaccines against pertussis, Hib, hepatitis B, invasive pneumococcal disease, IMD due to MenC, measles, mumps and rubella.

There will be no penal sanctions or fines for those who will not comply with the mandate but no exemption other than on medical grounds will be accepted. The French policy will therefore be comparable to that in place in the United States (US) since decades. One major difference is the possibility in many US states to ‘escape’ the mandates through exemptions for either philosophical or religious reasons. It has been shown, however, that such exemptions lead to sub-optimal control of vaccine preventable diseases and the current trend is to move away from those exemptions, as illustrated by the Californian or the Australian examples [16-18].

Issues related to philosophical exemptions have been analysed by the Ministry of Health, which dismissed this possibility in France. Indeed the French Constitutional Council, in a case between the State and a family that refused to administer the three mandatory vaccinations to their child, concluded in 2015 that ‘the legislator (through the vaccination mandates) did not undermine the constitutional requirement of health protection as guaranteed by the Preamble of 1946’. The reduction of freedom of choice was justified by the public health imperative of having every child immunised, consequently only public health considerations (namely medical contraindications) can justify an exemption.

## Challenges posed by the mandates

A legitimate concern raised by some is that this measure may be counterproductive. In a letter to the journal *Science*, Ward et al. made the prediction that ‘… *making more vaccine mandatory will convert vaccine hesitancy into a more extreme anti-vaccination stance.*’ and an editorial comment in *Nature* concluded that the *“heavy-hand law…could fuel further unfounded resistance to life-saving vaccines*” [19,20]. That the law will displease the anti-vaccination movement is certain. That it will make the hesitant become resistant is much more hypothetical. On the contrary, the extension of the mandates should contribute to restoring confidence rather than undermining it. It may well be perceived by a large majority of the French population as a strong and positive signal of the government’s commitment in favour of vaccination. It should reassure the population and the health professionals that the Ministry of Health considers the current recommended vaccines as necessary, effective and safe as those already mandatory. Of note, in a survey conducted in 2006, on a representative sample of more than 4,000 adults, less than 10% answered that they were in disfavour of vaccination mandates [21].

That the new law is merely an extension of the already implemented vaccination mandates for several infant vaccinations, will greatly facilitate its enforcement in real life. Showing proof of DTP immunisation for all forms of socialisation of children (nurseries, pre-schools, schools or any leisure activities) is part of the routine registration procedure and is well accepted both by the population and the services for children. Legal appeals made by parents after their DTP unimmunised child was denied admission to a community of children have been exceptional and unsuccessful.

## Tackling vaccine hesitancy

This new policy is intended to be temporary for the period needed to restore the confidence of the general public. Regular surveys monitoring trends in vaccine confidence will be conducted by Santé publique France to guide a decision if and when the mandates may be considered as no more necessary to maintain a high vaccination coverage.

However, the mandates by themselves will not solve the issue of the growing vaccine hesitancy. This will require a comprehensive social marketing and communication strategy. The ministry of Health and Public Health France will conduct, starting in 2018, large scale actions to promote vaccinations towards the general public, and to better support healthcare professionals faced with the hesitation of their patients. As a first step, in April 2017, Santé publique France launched a governmental website, *vaccination-info-service.fr*, that provides state-of-the art-information on all aspects of vaccines and vaccination. So far, over 2.5 million individuals have visited the website, confirming the demand of the population for transparent, authoritative and evidence-based information.

To restore confidence in vaccination beyond the parents of young children it is even all the more necessary that the expected increase in infant vaccination coverage will be insufficient, at least in the short term, to control measles or IMD due to MenC. A meta-analysis of meningococcal carriage data concluded that adolescents and young adults are the main reservoir of the pathogen [22]. For measles, seroprevalence and epidemiological data showed that in France, apart from infants less than one year of age, the main susceptibility is with adolescents and young adults [8,9]. This is the consequence of the insufficient initial coverage and catch-up in the cohorts born in the 1980s.

The new law only targets new birth cohorts. It will therefore take years to have a significant impact on the epidemiology of measles and MenC, unless vaccine coverage rapidly increases in older birth cohorts. One of the main challenges will be to convince all stakeholders that vaccination of older children, including boosters or catching up of vaccinations and the human papillomavirus (HPV) vaccination of adolescent girls, are as important as vaccinations in early childhood, although they remain only recommended.

Why were the vaccination mandates restricted to children below 2 years of age rather than including later boosters and catch up strategies? First, as the French Constitutional Council has justified the compulsory nature of vaccinations in children up to the age of 2 years by the medical and Public Health imperative, it was felt that making pre-school or school-age DTP boosters mandatory could not be justified on similar grounds. Contrary to the non-compliance with primary vaccinations, epidemiologists could not provide data to show that non-compliance to the DTP boosters recommended at 5 and 12 years would pose a threat for children concerned or society as a whole. More importantly, extension of the mandates to this age group would probably result in huge implementation challenges. As already mentioned, checking of the DTP immunisation status before registration into any collective child institution (leading to virtually 100% coverage for mandatory vaccination at pre-school entry) is well accepted by the French society. Making all children and adolescent vaccinations mandatory would have implied to exclude children with non-up-to-date immunisation status from preschool and school. This was considered neither socially acceptable nor feasible. In addition it would have much likely fuelled the anti-vaccination movement.

The extension of the vaccination mandates, restricted to new birth cohorts, is only one element of a much more ambitious endeavour, which is to strengthen the French vaccination programme.

The strong commitment of the current Minister of Health that resulted in the change in legislation and the wide support of GPs, paediatricians and infectious diseases learned societies for the extension of vaccination mandates, as assessed by their public position statement [23], should be the starting point of a series of actions that will help restore confidence in vaccines.

## Conclusion

France has an excellent vaccination coverage for compulsory vaccinations in early childhood but a largely insufficient coverage for recommended ones, and there is increasing vaccine hesitancy. The lifting of vaccination obligations would have entailed the risk of reducing the very high coverage of the mandatory vaccinations without improving it for the insufficient ones. Conversely, the extension of the immunisation obligations gives a strong positive signal to the general public and health professionals about the importance that the government gives to the protection of all children against severe diseases and should reassure them about the effectiveness and safety of all vaccines included in the routine immunisation schedule in early childhood. The increase in vaccination coverage that it will likely induce in future birth cohorts should allow, if the new mandates are actually enforced and associated with a catch-up in the older ones, a better control and even the elimination of vaccine preventable diseases still responsible today for an unacceptable human toll in France.
